# The Use of Probiotics to Fight Biofilms in Medical Devices: A Systematic Review and Meta-Analysis

**DOI:** 10.3390/microorganisms9010027

**Published:** 2020-12-23

**Authors:** Fábio M. Carvalho, Rita Teixeira-Santos, Filipe J. M. Mergulhão, Luciana C. Gomes

**Affiliations:** LEPABE—Laboratory for Process Engineering, Environment, Biotechnology and Energy, Faculty of Engineering, University of Porto, Rua Dr. Roberto Frias, 4200-465 Porto, Portugal; up201502963@fe.up.pt (F.M.C.); ritadtsantos@fe.up.pt (R.T.-S.); filipem@fe.up.pt (F.J.M.M.)

**Keywords:** probiotics, biofilm formation, antibiofilm strategies, medical device surface

## Abstract

Medical device-associated infections (MDAI) are a critical problem due to the increasing usage of medical devices in the aging population. The inhibition of biofilm formation through the use of probiotics has received attention from the medical field in the last years. However, this sparse knowledge has not been properly reviewed, so that successful strategies for biofilm management can be developed. This study aims to summarize the relevant literature about the effect of probiotics and their metabolites on biofilm formation in medical devices using a PRISMA-oriented (Preferred Reporting Items for Systematic reviews and Meta-Analyses) systematic search and meta-analysis. This approach revealed that the use of probiotics and their products is a promising strategy to hinder biofilm growth by a broad spectrum of pathogenic microorganisms. The meta-analysis showed a pooled effect estimate for the proportion of biofilm reduction of 70% for biosurfactants, 76% for cell-free supernatants (CFS), 77% for probiotic cells and 88% for exopolysaccharides (EPS). This review also highlights the need to properly analyze and report data, as well as the importance of standardizing the in vitro culture conditions to facilitate the comparison between studies. This is essential to increase the predictive value of the studies and translate their findings into clinical applications.

## 1. Introduction

Medical devices have been widely used in the prevention, diagnosis and treatment of some diseases, improving the healthcare and life quality of patients [1,2,3]. However, indwelling medical devices, such as mechanical heart valves, artificial veins or catheters, are particularly susceptible to microbial contamination [4,5,6], and their colonization poses a critical problem in the increasing number of healthcare-associated infections (HCAI) [7,8,9]. These have been associated with high mortality and morbidity rates, increased length of hospital stay and increased cost of treatment [2,8,10]. Medical device-associated infections (MDAI) comprise 50–70% of all HCAI [2,11]. MDAI are mostly originated from the formation of pathogenic biofilms on the device surface [1,12,13,14]. The increasingly widespread ability of pathogens to generate persistent biofilms and the low efficiency of the human immune system and antibiotics to counteract biofilm development are the base of recurrent biofilm-related infections in medical devices [2,5,15,16]. Biofilms formed on medical devices may be composed of Gram-positive and Gram-negative bacteria and yeasts [1,2,10,14]. They are defined as communities of microorganisms protected by a self-synthesized matrix of extracellular polymeric substances [17,18,19]. The extracellular matrix usually includes exopolysaccharides (EPS), proteins and nucleic acids [3,17,20], and protects the pathogens against host defense and antimicrobial agents by limiting the diffusion of antibiotics [17,21,22], enhancing the horizontal transmission of plasmid-associated antibiotic-resistant genes and creating an altered microenvironment [3,23]. Cells in biofilms are 10 to 1000 times more resistant to antimicrobial treatments than their planktonic counterparts [24]. Thus, the development of biofilms causes numerous problems in the biomedical field and constitutes a challenge in treating MDAI.

**Figure 1:**
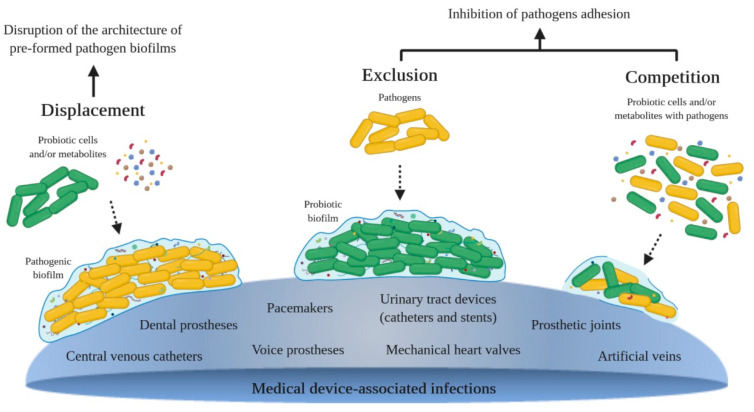
Antibiofilm strategies of probiotic cells and/or their metabolites: displacement–probiotics and/or their substances are added to a pre-formed pathogen biofilm; exclusion–pathogenic cells are added to a pre-formed probiotic biofilm or to a surface pre-coated with antibiofilm substances isolated from probiotics; competition–probiotic cells and/or their substances and pathogenic cells are co-cultured.

Novel technologies to prevent biofilm formation on medical devices, such as bactericidal coatings and adhesion-resistant surfaces, are being developed [8,25]. The increasing evidence of the effect of probiotics on the prevention and treatment of device-associated biofilms, and an increasing interest in promoting natural approaches to health have intensified the research in the field of probiotics and their metabolites to battle pathogenic biofilms [1,14,26,27,28,29]. Probiotics are defined as non-pathogenic live microorganisms (bacteria or yeasts) that, when administered in appropriate amounts, produce health benefits on the host. They have been used in clinical practice, mainly to restore the balance of the gastrointestinal tract [30,31,32,33]. Probiotics have received substantial attention regarding their health-promoting properties, possessing the status of Generally Regarded as Safe (GRAS) [34,35]. The most commonly used probiotics are species of lactic acid bacteria (LAB), which include *Lactobacillus*, *Bifidobacterium*, *Streptococcus*, *Lactococcus* and *Leuconostoc* [35,36,37]. Probiotics may act by displacement, exclusion and competition with pathogenic bacteria (Figure 1). The displacement strategy consists in the disruption of the architecture of pre-formed pathogen biofilms through the addition of probiotics and/or their metabolites; exclusion consists in pre-coating a surface with probiotics and/or their metabolites in order to inhibit pathogen adhesion; and competition consists in the co-culture of probiotics and/or their metabolites and pathogenic cells [34,38,39,40,41,42]. The main antimicrobial substances produced by probiotic cells are organic acids (lactic, acetic, propionic and succinic acid), hydrogen sulfide and peroxide, ethanol, carbon dioxide, EPS, biosurfactants and bacteriocins [34,35,38,39,41,43,44,45,46,47,48].

In this work, the currently available data regarding the potential of using probiotics to fight biofilm formation in medical devices were systematically reviewed. To the best of our knowledge, this is the first systematic review with meta-analysis about the anti-adhesive and antimicrobial activity of probiotics against medical device-associated infections.

## 2. Methods

### 2.1. Search Strategy, Study Eligibility and Data Extraction

Previously published studies on the use of probiotics for the control and prevention of biofilm formation in medical devices were systematically reviewed according to a PRISMA Statement [49]. The search was carried out until 7 April 2020 using the following databases: PubMed, ScienceDirect, Cochrane Library and Compendex. The search strategy combined a set of central keywords—Probiotic, Biofilm, Surface and Medical devices—with a wide range of terms and their combinations. Moreover, the reference sections of all included articles and screened reviews were hand-searched for additional articles that were not identified through the database search. The search was limited to articles published from 1980 to April 2020 in English language only.

Peer-reviewed full-text articles were then assessed for eligibility. The inclusion criteria were: (1) studies where probiotic cells and/or substances resulting from their metabolism are used as a way to control pathogens; (2) inhibition strategies of pathogens, including displacement, exclusion and competition. The exclusion criteria consisted of: (1) studies focused on the antimicrobial effect of probiotics and/or substances isolated from them without assessing their antibiofilm potential; (2) studies where biotic surfaces such as epithelial tissues are used as substratum; and (3) non-original articles (including reviews or reports).

Information regarding the inhibition strategy of pathogens, probiotic strains and/or their antibiofilm substances, biofilm-forming pathogens, surface material, used methodologies (including culture conditions, biofilm platforms and biofilm techniques) and obtained outcomes were extracted from each included study and inserted in an electronic spreadsheet. Posteriorly, this data was confirmed by another reviewer. Moreover, the percentage of reduction of biofilm formation was retrieved whenever possible for meta-analysis. If this result was not described, an estimate was made with the values obtained from graphs and tables and compared with a control.

### 2.2. Quality Assessment

The quality assessment of the selected studies was conducted according to an adapted Methodological Index for Non-Randomized Studies (MINORS) scale [50]. MINORS is a validated instrument designed to assess the methodological quality and potential bias of non-randomized surgical studies [50]. Although there are no methodological indices to measure the risk or the quality of laboratory-based studies, the MINORS scale was adapted to this specific context to assess whether the studies are representative of real conditions [51], evaluating their predictive value. The following parameters were considered: (1) a clearly stated aim; (2) detection of bias; (3) an adequate control group; (4) an appropriate methodology; (5) description of pathogens, (6) antibiofilm substances, (7) culture conditions, (8) biofilm formation period and (9) surface substratum; (10) the predictive value of the study; (11) clarity of results and (12) adequate statistical analyses. Like the original MINORS scale, the modified scale consists of 12 items scored on a 3-point scale: 0 (not reported), 1 (inadequately reported) or 2 (adequately reported), where the ideal global score would be 24.

### 2.3. Meta-Analysis

The meta-analysis was performed according to Harrer and co-workers’ methodology for meta-analysis [52]. The packages “meta” and “metafor” for R programming language were used to estimate the pooled effect sizes, heterogeneity testing and funnel plotting. The individual studies’ effect estimates were retrieved from the reported proportions of biofilm reduction. The heterogeneity between studies was evaluated using the *I*^2^ and *τ*^2^ tests. A *p*-value < 0.05 was considered statistically significant, and *I*^2^ ≥ 50% indicates the existence of significant heterogeneity, while *τ*^2^ equal to zero or close to zero indicates that there is no variance between studies [52,53,54]. The publication bias of the selected studies was assessed using Begg’s funnel plot and Egger’s test [55].

## 3. Results and Discussion

### 3.1. Study Selection and Characterization

**Figure 2:**
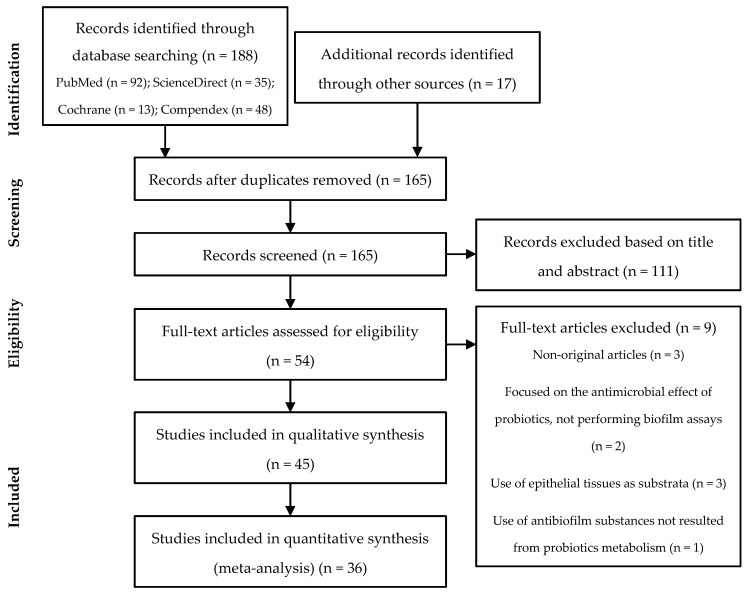
Preferred Reporting Items for Systematic reviews and Meta-Analyses (PRISMA) flow-chart.

The search resulted in a total of 188 articles identified through database searching using the described methodology. This number was increased upon the inclusion of 17 additional records identified through other sources (previous searches and references of selected articles), resulting in a total of 205 studies. After duplicates removal, 165 records proceeded to the screening phase. From these, 111 records were excluded based on the title and abstract, since they did not fulfill the pre-determined criteria for eligibility. Further examination of the remaining 54 full-text articles resulted in the exclusion of 9 articles according to the exclusion criteria: 2 studies were focused on the antimicrobial effect of probiotics, not performing biofilm assays; 3 studies used epithelial tissues as substrata; 1 study used antibiofilm substances not resulting from probiotics metabolism; 3 studies correspond to non-original articles. Of the 45 studies eligible for qualitative synthesis, 36 presented the required data for meta-analysis. All this information is schematized in Figure 2.

Currently, the treatment of biofilm-related infections still depends on conventional antimicrobial therapies, which increases selective pressure in favor of antibiotic-resistant strains, posing a threat to patients’ health [2,6]. Probiotics have recently been considered as a reliable option to inhibit or delay the onset of biofilm formation on medical devices [56]. In this context, the main advances on probiotics and their metabolites for preventing and eradicating biofilms from the surfaces are reviewed and discussed. Table 1, Table 2 and Table 3 divide the studies into the three antibiofilm strategies presented in Figure 1 (displacement, exclusion and competition, respectively) and show the effect of probiotics and their metabolites against several bacterial and fungal species. The division into different strategies facilitates the identification of which of the stages of biofilm development is more suitable for a probiotic-based control strategy. On the other hand, some antibiofilm substances may be more compatible with a particular strategy due to their intrinsic characteristics. Thus, studies within each strategy were grouped according to the antibiofilm substance, and all strategies included the use of biosurfactants, bacteriocins, EPS, cell-free supernatants (CFS, except for the exclusion strategy), cells, and other less representative substances isolated from probiotics. Because one article can hold more than one strategy and/or antibiofilm substance, a total of 22 experiments were included for displacement, 23 for exclusion and 33 for the competition strategy. The most used methodologies for biofilm examination were CFU (colony-forming units) counting and crystal violet (CV) staining, which were used in 23 and 22 studies of the total 45, respectively. Polystyrene and silicone-based surfaces were the most used material (33% and 31% of the studies, respectively). Moreover, this review addresses microbial biofilms developed on a wide range of indwelling medical devices, such as central venous catheters [57], urinary tract devices (catheters and stents) [12,58,59], voice prostheses [60,61,62], and dental prostheses [63,64,65]. Regarding the antibiofilm substances, probiotic cells (44% of the studies) were the most used, followed by biosurfactants (24% of the studies), and *Lactobacillus* was the dominant probiotic genus.

Biosurfactant production is a mechanism by which probiotics interfere with pathogens. It has been shown that the adsorption of biosurfactants to a substratum may interfere with microbial adhesion and desorption processes [66,67]. Biosurfactants are a structurally diverse group of surface-active compounds released or associated with the cell wall of a wide variety of microorganisms [68,69,70], with amphipathic properties (both hydrophilic and hydrophobic moieties within the same molecule) [57,71,72]. Biosurfactants can be classified according to their chemical structure in glycolipids (e.g., rhamnolipids, trehalolipids and sophorolipids), lipoproteins or lipopeptides (e.g., surfactin), phospholipids, fatty acids or natural lipids (e.g., gramicidin and polymixin), polymeric biosurfactants (e.g., emulsan, alasan and liposan) and particulate biosurfactants [66,73,74,75]. Recently, biosurfactants have received special attention due to their advantages over synthetic surfactants, such as higher biodegradability, lower toxicity and effectiveness in extreme environments [67,69,70,76]. Depending on their molecular weight, the most common methods used for extraction and purification of biosurfactants include precipitation (with acid, aluminium sulphate or ammonium sulphate), solvent extraction, membrane filtration, centrifugation, selective crystallization, foam fractionation and chromatographic techniques [73,74,75,77,78]. Although the mechanisms of action of biosurfactants are not fully elucidated, due to their amphipathic nature, they can form an interfacial film that affects the properties (surface energy and wettability) of the original surface, modifying its hydrophobicity, reducing the surface and interfacial tensions, and thus affecting the cell attachment and enhancing desorption [66,67,79,80]. Additionally, biosurfactants may have a bactericidal behavior by disrupting the cytoplasmic membrane of pathogens or by disturbing protein conformations, causing cell lysis and metabolite leakage [57,81,82]. The interactions of biosurfactants with cell membranes are related to their insertion into the lipid bilayers, chelation of cations, channel formation and membrane solubilization, that compromise the bilayer stability [67]. Moreover, biosurfactants may perturb the cell division process [27], as well as affect the expression of biofilm-related genes, thereby interfering with the release of signaling molecules in quorum sensing (QS) systems and the subsequent biofilm formation [70,83].

Several studies have reported the effectiveness of biosurfactants from probiotics in antagonizing microbial biofilm formation on abiotic surfaces. Biosurfactants were studied on different surfaces, including silicone-based surfaces, polystyrene, polydimethylsiloxane (PDMS) and titanium discs, and tested against a broad spectrum of Gram-positive and Gram-negative bacteria and yeasts. The antibiofilm properties of biosurfactants were examined in the three strategies; however, due to the tendency of these molecules to accumulate at the interfaces and change the surface tension and hydrophobicity, the majority of the studies inspected the pre-conditioning of the surface materials with these substances.

**Table 1:** Displacement studies in medical devices.

Antibiofilm Substances and Probiotic Strains		Abiotic Surface	Biofilm Forming Pathogens	Percentages of Reduction	Ref.	Major Conclusions
Biosurfactants	*L. brevis* *L. gasseri* *L. jensenii* *L. rhamnosus*	Polystyrene Silicone elastomeric discs	*Ac. baumannii* *C. albicans* *C. krusei* *C. tropicalis* *En. aerogenes* *E. coli* *K. pneumoniae* *S. aureus* *S. saprophyticus*	58% 37% 37% 33% 64% 46%–65% 16% 61% 39%	[27] [57,80] [80] [80] [80] [27,80] [80] [27] [80]	Biosurfactants disrupted the biofilms of all bacteria by 16%–65%, depending on the concentration. For yeasts, a biofilm reduction of 35% was achieved.
Bacteriocins	*L. acidophilus* *L. plantarum*	Foley silicone catheter pieces Polystyrene	*P. aeruginosa* *Ser. marcescens*	59% 48%	[84] [85]	Bacteriocins showed inhibitory activity against *P. aeruginosa* (59%) and living cells of *Ser. marcescens* (48%).
EPS	*Leu. citreum* *Leu. mesenteroides* *Leu. pseudo-mesenteroides* *Ped. pentosaceus*	N.A.	*Ent. faecalis* *E. coli* *S. aureus*	53% 62% 77%	[86] [86] [86]	The capacity of EPS to disrupt pre-formed biofilms increased with increasing concentrations, and it was lower than the capacity to prevent adhesion. Biofilm formation was reduced by 53%–77%.
Cell-free supernatants	*L. fermentum* *L. gasseri* *L. helveticus* *L. pentosus* *L. plantarum* *L. rhamnosus* *Strep. salivarius*	Glass Polystyrene Polyurethane PVC	*C. albicans* *C. krusei* *C. parapsilosis* *C. tropicalis* *E. coli* *K. pneumoniae* *P. aeruginosa* *S. aureus*	80% 67% 40% 64% N.A. 78% 74% 50%	[87] [88] [88] [88] [89] [90] [90] [91]	CFS induced biofilm disruption on the different surfaces by 38%–80%, depending on the species. The neutralized supernatants inhibited *P. aeruginosa* (74%) and *K. pneumoniae* biofilm formation (78%).
Cells	*B. infantis* *B. longum* *Ent. faecium* *L. acidophilus* *L. casei* *L. casei rhamnosus* *L. casei shirota* *L. fermentum* *L. helveticus* *L. paracasei* *L. plantarum* *L. reuteri* *L. rhamnosus* *L. rhamnosus* GG *Lact. lactis* *Lact. lactis cremoris* *Strep. cremoris* *Strep. salivarius* *Strep. thermophilus*	Bovine enamel saliva-coated Denture surface Glass Polyurethane Saliva-conditioned titanium discs Silicone latex Silicone rubber	*At. vaginae* *C. albicans* *C. tropicalis* *E. coli* *G. vaginalis* *S. aureus* *Strep. mutans* *Strep. oralis* Staphylococcal strains Streptococcal strains	N.A. 80%–99% 88%–95% 80% N.A. 98% 29%–99% 99% 83% 83%	[89] [60,65,87,92] [60,92] [93] [89] [94] [95,96] [96] [60] [60]	Probiotics overlaid on pre-formed biofilms reduced the biofilm culturable cells of Gram-positive bacteria by 79%–99% and biofilm formation by 89%–94%. Biofilm culturable cells of yeasts were reduced by more than 63%. *B. infantis* and *Ent. faecium* did not reduce the number of yeasts in biofilms. *L. rhamnosus* microcapsules reduced *E. coli* culturable cells in the biofilm up to 80%, in a dose-dependent manner.
Lipoteichoic acid (LTA)	*L. plantarum*	Glass Polystyrene	*A. naeslundii* *Ent. faecalis* *L. salivarius* *Strep. mutans*	N.A. N.A. N.A. N.A.	[97] [97] [97] [97,98]	LTA activity was inconsistent.

Abbreviations: CFS, Cell-Free Supernatant; EPS, Exopolysaccharides; PVC, Polyvinyl Chloride; N.A., Not Available. *Ac*., *Acinetobacter*; *A*., *Actinomyces*; *At*., *Atopobium*; *B*., *Bifidobacterium*; *C*., *Candida*; *En*., *Enterobacter*; *Ent*., *Enterococcus*; *E*., *Escherichia*; *G*., *Gardnerella*; *K*., *Klebsiella*; *L*., *Lactobacillus*; *Lact*., *Lactococcus*; *Leu*., *Leuconostoc*; *Ped*., *Pediococcus*; *P*., *Pseudomonas*; *Ser*., *Serratia*; *S*., *Staphylococcus*; *Strep*., *Streptococcus*.

**Table 2:** Exclusion studies in medical devices.

Antibiofilm Substances and Probiotic Strains		Abiotic Surface	Biofilm Forming Pathogens	Percentages of Reduction	Ref.	Major Conclusions
Biosurfactants	*L. acidophilus* *L. brevis* *L. casei* *L. delbrueckii* *L. fermentum* *L. helveticus* *L. paracasei* *L. plantarum* *L. reuteri* *L. rhamnosus* *Lact. lactis* *Strep. thermophilus*	PDMS discs Polystyrene Silicone elastomeric discs Silicone rubber	*Bac. cereus* *Bac. subtilis* *C. albicans* *C. tropicalis* *Ent. faecalis* *E. coli* *K. pneumoniae* *Lis. innocua* *Lis. monocytogenes* *Pr. mirabilis* *Pr. vulgaris* *Prov. stuartii* *P. aeruginosa* *P. putida* *R. dentocariosa* *Sal. typhi* *Ser. marcescens* *Sh. flexneri* *S. aureus* *S. epidermidis* *Strep. salivarius*	87% 79% 50%–85% 56%–67% N.A. 50%–59% N.A. 82% 84% N.A. 65%–75% N.A. 49%–70% 65% 78%–89% 56% 60% 40% 61%–96% 85%–94% 90%–93%	[81] [99] [57,62,100,101] [61,62] [101] [81,99,101] [101] [81] [81] [101] [99,100] [101] [81,99,101] [99] [61,62] [81] [102] [81] [61,62,81,99,100] [61,62,81,101] [61,62]	Pre-adsorbed biosurfactants displayed high anti-adhesive activity against both Gram-positive (61–97%) and Gram-negative (40%–75%) bacteria. Pre-adsorbed biosurfactant reduced the adhesion of yeasts to silicone by 50%–85%.
Bacteriocins	*L. fermentum* *L. plantarum*	Foley silicone catheter pieces Polystyrene	*P. aeruginosa* *S. aureus*	99% N.A.	[103] [59,103]	Pre-coating with bacteriocins reduced the number of biofilm culturable cells by 99%.
EPS	*L. fermentum* *Leu. citreum* *Leu. mesenteroides* *Leu. pseudo-mesenteroides* *Ped. pentosaceus*	Polystyrene	*Ent. faecalis* *E. coli* *P. aeruginosa* *S. aureus*	88% 90% 96% 87%	[86] [86] [103] [86]	Pre-coating with EPS reduced the number of biofilm culturable cells of *P. aeruginosa* by 96% and inhibited the adhesion of bacteria in a dose-dependent manner (87%–90%).
Cells	*E. coli* Nissle 1917 *L. acidophilus* *L. casei* *L. casei rhamnosus L. fermentum* *L. paracasei* *L. rhamnosus* *Lact. lactis* *Lact. lactis *ssp. *lactis* *Strep. thermophilus*	Denture surface Foley silicone catheter pieces Glass Saliva-coated hydroxyapatite discs Polystyrene Saliva-conditioned titanium discs Silicone Silicone latex	*A. naeslundii* *C. albicans* *Ent. faecalis* *E. coli* *F. nucleatum* *Klebsiella* ssp. Non-mutans streptococci strains *P. aeruginosa* *S. aureus* *Strep. mutans* *Strep. oralis* *Strep. sobrinus* *V. dispar*	33% 99% 99% 99% 60% N.A. 8% N.A. 99% 30%-99% 79%-99% 89% 68%	[104] [65] [12] [105] [104] [105] [106] [105] [94,105,107] [96,106] [96,104] [104] [104]	Probiotics reduced the adhesion of pathogens up to 3 Log CFU and biofilm biomass by 8%–30%. Pre-coating with EcN biofilms reduced the adherence of *Ent. faecalis* on silicone up to 2 Log CFU.
Collagen-binding protein (p29)	*L. fermentum*	Polyisobutylene-polystyrene (PIB-PS) copolymerSilicone rubber	*Ent. faecalis* *E. coli*	47% 75%	[58] [58]	Coating with p29 resulted in a reduction of 34% and 75% in *E. coli* adhesion, and 47% and 18% in *Ent. faecalis* adhesion to silicone rubber and PIB-PS, respectively.
Lipoteichoic acid (LTA)	*L. plantarum*	Polystyrene	*Strep. mutans*	40%	[98]	Biofilm formation was inhibited, but to a lesser degree in comparison with co-incubation.

Abbreviations: CFU, Colony-Forming Units; EcN, *E. coli* Nissle 1917; EPS, Exopolysaccharides; PDMS, Polydimethylsiloxane; N.A., Not Available. *A*., *Actinomyces*; *Bac*., *Bacillus*; *C*., *Candida*; *Ent*., *Enterococcus*; *E*., *Escherichia*; *F*., *Fusobacterium*; *K*., *Klebsiella*; *L*., *Lactobacillus*; *Lact*., *Lactococcus*; *Leu*., *Leuconostoc*; *Lis*., *Listeria*; *Ped*., *Pediococcus*; *Pr*., *Proteus*; *Prov*., *Providencia*; *P*., *Pseudomonas*; *R*., *Rothia*; *Sal*., *Salmonella*; *Ser*., *Serratia*; *Sh*., *Shigella*; *S*., *Staphylococcus*; *Strep*., *Streptococcus*; *V*., *Veillonella*.

**Table 3:** Competition studies in medical devices.

Antibiofilm Substances and Probiotic Strains		Abiotic Surface	Biofilm Forming Pathogens	Percentages of Reduction	Ref.	Major Conclusions
Biosurfactants	*L. acidophilus* *L. brevis* *L. helveticus* *L. jensenii* *L. paracasei* *L. reuteri* *L. rhamnosus*	Medical grade silicone tubes Polystyrene Polystyrene pre-coated with human plasma Silicone elastomeric discs Saliva-conditioned titanium discs	*Ac. Baumannii* *Bac. cereus* *C. albicans* *E. coli* *P. aeruginosa* *Ser. marcescens* *S. aureus* *Strep. mutans* *Strep. oralis*	76% 100% 90%–100% 79%–100% 100% 73% 88%–100% 99% 99%	[27] [81] [57,81] [27,81] [81] [102] [27,81] [64] [64]	Biosurfactants displayed high anti-adhesive activity and reduced biofilm biomass by 60%–100% and culturable cells by 90%–99%. The inhibitory effect was dose-dependent.
Bacteriocins	*L. fermentum* *L. plantarum*	Polystyrene	*P. aeruginosa* *S. aureus*	56%–93% 62%	[59,103] [59]	Co-incubation with bacteriocins reduced the number of *P. aeruginosa* culturable cells by 93% and biofilm formation of pathogens by 56%–62%.
EPS	*L. delbrueckii* ssp. *bulgaricus* *L. fermentum* *L. rhamnosus*	Polystyrene	*Bac. cereus* *Ent. faecalis* *Lis. monocytogenes* *P. aeruginosa*	90% 87% 88% 88%–97%	[108] [108] [108] [103,108]	Co-incubation with EPS reduced the number of *P. aeruginosa* culturable cells by 97% and inhibited biofilm formation between 74% and 90%, depending on the species, in a dose-dependent manner.
Cell-free supernatants	*Bac. subtilis* *L. acidophilus* *L. fermentum* *L. gasseri* *L. helveticus* *L. paracasei* *L. plantarum* *L. rhamnosus* *Strep. salivarius*	Glass Polystyrene Polyurethane PVC Saliva-conditioned titanium discs Silicone	*C. albicans* *C. krusei* *C. parapsilosis* *C. tropicalis* *Ent. faecalis* *E. coli* *K. pneumoniae* *P. aeruginosa* *S. aureus* *Strep. mutans* *Strep. oralis*	90% 71% 41% 67% 61% 63% 99% 57% 57%–99% 53%–99% 99%	[87] [88] [88] [88] [109] [109] [110] [91] [91,111] [96,98,112] [96]	CFS were able to reduce the number of biofilm cells by more than 81% and inhibit the ability of pathogens to adhere to the different surfaces by 39–99%. Neutralized supernatants had less effect on biofilm formation.
Cells	*E. coli* Nissle 1917 *L. acidophilus* *L. casei* *L. casei rhamnosus* *L. fermentum* *L. helveticus* *L. paracasei* *L. plantarum* *L. rhamnosus* *L. rhamnosus* GG *L. salivarius* *Lact. lactis* ssp. *lactis* *Strep. thermophilus*	Bovine enamel saliva-coated Glass Polystyrene Polyurethane Polypropylene Saliva-coated hydroxyapatite discs Saliva-conditioned titanium discs Silicone latex Silicone rubber	*A. naeslundii* *C. albicans* *E. coli* *F. nucleatum* *K. pneumoniae* Non-mutans streptococci strains *P. aeruginosa* *S. aureus* *S. epidermidis* *Strep. mutans* *Strep. oralis* *Strep. sanguinisStrep. sobrinus* *V. díspar*	22% 53%–72% 82%–93% 55% 99% 11% N.A. 99% 99% 9%–99% 65%–99% N.A. 76% 32%	[104] [63,87] [93,113,114] [104] [110] [106] [113] [94,113] [113] [63,95,96,106,112] [96,104] [63] [104] [104]	The adhesion of pathogens was reduced by the presence of probiotic cells (11%–93%), and their culturability decreased up to 7.2 Log CFU. *L. rhamnosus* microcapsules reduced biofilm formation up to 82% in a dose-dependent manner. *Lactobacillus* strains inhibited the growth of an uropathogenic biofilm on silicone rubber for at least 8 days. EcN was able to outcompete pathogenic strains during biofilm formation, reducing culturability up to 4 Log.
Lipoteichoic acid (LTA)	*L. plantarum*	Glass Human dentin slices Polystyrene Saliva-coated hydroxyapatite discs	*A. naeslundii* *Ent. faecalis* *L. salivarius* *Strep. mutans*	57% 57% 57% 57%–75%	[97] [97] [97] [97,98]	LTA inhibited single- and multi-species biofilm formation by 75% and 57%, respectively.

Abbreviations: CFS, Cell-Free Supernatant; CFU, Colony-Forming Units; EcN, *E. coli* Nissle 1917; EPS, Exopolysaccharides; PVC, Polyvinyl Chloride; N.A., Not Available. *Ac*., *Acinetobacter*; *A*., *Actinomyces*; *Bac*., *Bacillus*; *C*., *Candida*; *Ent*., *Enterococcus*; *E*., *Escherichia*; *F*., *Fusobacterium*; *K*., *Klebsiella*; *L*., *Lactobacillus*; *Lact*., *Lactococcus*; *Lis*., *Listeria*; *P*., *Pseudomonas*; *Ser*., *Serratia*; *S*., *Staphylococcus*; *Strep*., *Streptococcus*; *V*., *Veillonella*.

Regarding the displacement strategy (Table 1), the data showed that different concentrations of biosurfactants were able to disrupt the biofilms of all tested bacteria on polystyrene at different levels (16%–65%) [27,80]. For yeasts, a biofilm reduction of about 35% was achieved [80]. When the same studies compared the effect of biosurfactants in displacement and other strategies, a lower reduction in biofilm formation was observed for the displacement strategy. Sambanthamoorthy et al. [27] demonstrated that biosurfactants reduced the initial adherence and disrupted pre-formed biofilms of clinical multidrug-resistant strains; however, their action was more pronounced on pre-coated surfaces than on pre-formed biofilms. Likewise, Ceresa et al. [57] demonstrated that biosurfactants decreased the initial deposition and biofilm growth of *Candida albicans* on silicone surfaces, but on pre-formed biofilms no significant inhibitory activity was observed. This suggests that biosurfactants are more suitable for the pre-coating and co-incubation approaches than for attacking pre-formed biofilms (displacement strategy).

Pre-adsorbed biosurfactants produced by *Lactobacillus* spp. strains (Table 2) showed good results in reducing the biofilm formation of both Gram-positive (61%–87%) and Gram-negative (40%–75%) bacteria on polystyrene [81,99,100,102]. Biosurfactants also demonstrated anti-adhesive potential against *Proteus vulgaris* and *Bacillus subtilis* on PDMS discs [99]. Anti-adhesive experiments on silicone rubber indicated their use as a promising strategy for the development of anti-adhesive biological coatings for urinary catheters [101] and voice prostheses [61,62]. Surlactin, a biosurfactant isolated from *Lactobacillus acidophilus* RC-14, caused a marked reduction in the adhesion of uropathogenic bacteria on silicone rubber after 4 h of flow [101]. Biosurfactants isolated from *Streptococcus thermophilus* A and *Lactococcus lactis* 53 significantly reduced the adhesion of *Staphylococcus epidermidis* and *Streptococcus salivarius* to silicone by more than 90% and of yeasts by 50%–85% [61,62].

Similarly, the results obtained with the competition strategy (Table 3) were very promising. All biosurfactants isolated from *Lactobacillus* spp. displayed high anti-adhesive activity and inhibited biofilm formation by a remarkable decrease in biomass production (60%–100%) and culturable cells (90%–99.9%). Biosurfactants were successfully tested on silicone-based surfaces, inhibiting almost completely the microbial adhesion after 3 days [57,81]. Likewise, biosurfactants presented high anti-adhesive action against the biofilm formation of *Serratia marcescens* (73%), *Acinetobacter baumannii* (76%), *Escherichia coli* (79%) and *Staphylococcus aureus* (88%) in polystyrene surfaces [27,102]. Sambanthamoorthy et al. [27] suggested that the structural differences in the cell wall and membranes observed between treated and untreated cells may be due to the biosurfactant interference in the cell division process. Additionally, Ciandrini et al. [64] showed that biosurfactants inhibited the adhesion and biofilm formation of *Streptococcus mutans* (77%–99.9%) and *Streptococcus oralis* (66%–98%) in saliva-conditioned titanium discs. Several studies demonstrated that this inhibitory effect in different biomedical scenarios is dose-dependent.

The applications of bacteriocins are expanding from the food industry to human health [115]. Bacteriocins are a heterogeneous group of ribosomal synthesized proteins or peptides with both bactericidal and bacteriostatic activities [115,116,117,118]. They are one of the most interesting alternatives to antibiotics due to their high stability, low toxicity, significant potency, and broad and narrow spectra of activity [59,116,118]. There are several proposed mechanisms for biofilm inhibition by bacteriocins: (1) pore formation in the target-cell wall, leading to cell leakage; (2) inhibition of cell wall synthesis; (3) depolarization of cytoplasmic membrane; and (4) permeabilization of the target-cell membrane, disrupting the proton motive force and causing cell death [26,39,115].

So far, few studies reported the in vitro effectiveness of bacteriocins produced by probiotics against biofilm formation on medical surfaces. Antibiofilm experiments were performed on silicone-based surfaces and polystyrene. Bacteriocins decreased the amount of pre-formed biofilms of *Pseudomonas aeruginosa* on Foley catheters by 59% [84] and of *Ser. marcescens* on polystyrene by 48% (Table 1) [85]. Likewise, Foley catheters coated with bacteriocins prevented the adhesion of *P. aeruginosa* and *S. aureus* (Table 2) [59]. Sharma et al. [103] compared the ability of bacteriocins to reduce the biofilm formation of *P. aeruginosa* PAO1 through pre-coating and co-incubation on polystyrene, and, in both experiments, the number of biofilm culturable cells was reduced by more than 93%. The same authors also demonstrated synergic associations between bacteriocins and EPS, which enhanced the death of *P. aeruginosa* (Table 2 and Table 3) [103]. Likewise, Mohapatra and Jeevaratnam [59] demonstrated that the co-incubation of bacteriocins and pathogens prevented the microbial adhesion of *P. aeruginosa* and *S. aureus* by 56% and 62%, respectively (Table 2 and Table 3). Although bacteriocins isolated from probiotics exhibited high antimicrobial activity, there are some limitations to their clinical application: (1) the development of resistance by pathogens upon continuous exposure to bacteriocins; (2) the low bioavailability, stability, half-life and solubility under physiological conditions, being susceptible to degradation by proteolytic enzymes; (3) the difficult purification, low yield and high production costs, decreasing the feasibility of large-scale production; (4) the existence of insufficient data on the safety and toxicity of bacteriocins; and (5) the lack of specific guidelines for legal approval of bacteriocins or bacteriocin-producing bacteria for medical applications [44,116,119,120,121]. Nevertheless, further investigation on improving the physicochemical and biological characteristics of bacteriocins can minimize those limitations.

In recent years, some bacterial EPS were proposed to control biofilm formation. EPS are a large group of long-chain high-molecular-mass biopolymers that are produced by the metabolic pathways of various microorganisms and differ in terms of monomer composition, molecular mass, degree of branching and structure [108,122,123,124]. Among the diversity of EPS-producing microorganisms, EPS from LAB have several applications due to their antioxidant, immunomodulating, anti-tumor and antimicrobial properties [122,123]. Although the EPS produced by probiotics have many industrial applications [123,124], their application as antibiofilm agents, particularly in the biomedical field, has been scarcely explored, and little is known about their mechanisms of action. However, it has been proposed that EPS may impair cell division, disrupting the cell wall and cytoplasmic membrane, and decomposing DNA molecules [103]. Moreover, EPS were suggested to interfere with *E. coli* biofilm formation by affecting genes related to chemotaxis and curli formation, and by reducing cell-cell interactions [125]. Yet, Mahdhi et al. [124] proposed that EPS decrease cell surface hydrophobicity and indole production (proposed as a signal molecule involved in QS), hindering *E. coli* biofilm formation, and inhibit the efflux pumps associated with bacterial adhesion and antimicrobial resistance.

EPS isolated from *Lactobacillus* spp. and *Leuconostoc* spp. presented anti-adhesive and antimicrobial activities against Gram-positive and Gram-negative bacteria in polystyrene. Their capacity to disperse biofilms was demonstrated against *E. coli*, *Enterococcus faecalis* and *S. aureus* (53% to 77% of biomass reduction) (Table 1) [86]. However, the ability of EPS to disrupt pre-formed biofilms is lower than their capacity to prevent adhesion. In fact, in both exclusion and competition strategies, EPS showed excellent anti-adhesive activity against all tested pathogenic biofilms, reducing biofilm formation by about 90% in a dose-dependent manner [86,108] and the number of biofilm culturable cells up to 97% (Table 2 and Table 3) [103]. These authors suggested that EPS have modified the matrix and inhibited the initial attachment and auto-aggregation of cells by affecting the bacterial surface properties and restricting cell-surface interactions [86,103]. Furthermore, EPS might have reduced the production of molecules involved in QS, whose function is to stimulate the expression of genes related to biofilm formation [103].

Probiotics are known to produce many metabolites with antimicrobial and antibiofilm activities, which are frequently secreted to the surrounding medium, such as bacteriocins and antimicrobial peptides, organic and fatty acids, biosurfactants and hydrogen peroxide [26,39]. These metabolites are often collected from the CFS, and thus the mechanism of action of supernatants is directly related to the antimicrobial metabolites produced by probiotics, which may differ among species.

In the last years, several studies using CFS were performed to determine their antibiofilm activity. The influence of CFS (mainly those isolated from *Lactobacillus* spp.) on biofilm formation was tested on different surfaces, including silicone, glass, polystyrene, polyurethane, polyvinyl chloride (PVC) and saliva-conditioned titanium discs, against a wide range of pathogens. Supernatants were not used in the exclusion strategy, probably due to limitations in forming a coating.

CFS induced biofilm disruption on different surfaces, between 38% and 80% reduction for *S. aureus*, *P. aeruginosa*, *Klebsiella pneumoniae* and *Candida* spp. biofilms (Table 1) [87,88,90,91]. The cell density of *E. coli* biofilms on glass also decreased when in contact with supernatants [89]. The activity of neutralized supernatants was assessed by Poornachandra et al. [90] to exclude the activity of organic acids. The supernatants showed good antibiofilm activity, inhibiting the biofilm formation of *P. aeruginosa* and *K. pneumoniae* up to 74% and 78%, respectively (Table 1 and Table 3), suggesting the presence of bioactive substances such as bacteriocins or biosurfactants. However, the ability of supernatants to prevent biofilm development seems to be higher than that of disrupting pre-formed biofilms. James et al. [87] and Varma et al. [91] compared the displacement and competition strategies in polystyrene and polyurethane, and PVC, respectively, and demonstrated that supernatants had a slightly higher effect in co-incubation assays (Table 1 and Table 3). Regarding the competition strategy (Table 3), CFS reduced the number of biofilm culturable cells by more than 81% [87,96,110,111] and inhibited pathogens adhesion to different surfaces by 39%–99% [87,88,91,96,98,109,110,111,112], depending on the species (Table 1 and Table 3). In addition, *Candida* spp. multi-species biofilm formation was reduced by 67% on silicone surfaces [88]. Likewise, supernatants were effective in reducing the number of culturable cells of oral biofilms of *Strep. oralis* and *Strep. mutans* by more than 5 Log CFU on saliva-conditioned titanium discs [96]. 

Similar to supernatants, the antibiofilm activity of the whole probiotic cell is directly related to the produced antimicrobial metabolites, and also with the competition for nutrients and adhesion sites on the surface [88]. It was noted that the action of probiotic cells was independent of the strategy adopted. In fact, when the same studies compared the effect of probiotic cells between the strategies, similar reductions were achieved either by pre-coating the surface, co-incubating or disrupting the pre-formed biofilms with probiotics [87,93,94,96,104,106]. The most tested probiotic species were *Lactobacillus rhamnosus*, *Lactobacillus plantarum*, *L. acidophilus* and *Lactobacillus fermentum*.

Probiotics were successfully tested on silicone-based surfaces, decreasing the amount of biofilm culturable cells of *S. aureus* (99.9%), *Ent. faecalis* (99.9%) and multi-species biofilms (83%–95%) (Table 1, Table 2 and Table 3), showing applicability in urinary catheters [12,94,107,126] and voice prostheses [60,92]. Moreover, probiotics exerted antibiofilm activity on polystyrene (Table 2 and Table 3), reducing the biofilm culturability of *E. coli* [93,105] and *K. pneumoniae* [110] up to 99.9%, and the biofilm amount of *Strep. mutans* strains between 28% and 70% [106,112]. Probiotic cells also reduced the number of biofilm culturable cells of *C. albicans* up to 80% in polyurethane (Table 1 and Table 3) [87], and of *E. coli* O157:H7, *S. aureus* and *S. epidermidis* up to 99.9% in polypropylene (Table 3) [113]. In glass, the introduction of *L. rhamnosus* and *Lactobacillus reuteri* into pre-formed biofilms resulted in a significant killing of pathogens (Table 1) [89,93], while the *L. acidophilus* coating demonstrated high resistance to bacterial adhesion (Table 2) [107]. Other applications where probiotics have been gaining interest are in the oral biofilm treatment and caries prevention. *L. rhamnosus* GG, *Strep. thermophilus* and *Lact. lactis* ssp. reduced the bacterial adhesion of several pathogens up to 85% in saliva-coated hydroxyapatite discs (Table 2 and Table 3) [63,104]. *Lactobacillus paracasei* and *L. rhamnosus* also evidenced a reduction between 2 and 8 Log CFU of *S. mutans* and *Strep. oralis* in saliva-conditioned titanium discs, depending on the strategy used (Table 1, Table 2 and Table 3) [96]. Likewise, *L. rhamnosus* GG and *Lactobacillus casei* disrupted and inhibited the formation of *C. albicans* biofilms on the denture surface by 99.9% (Table 1 and Table 2) [65]. Song et al. [93] demonstrated that the use of microcapsules containing *L. rhamnosus* GG cells disrupted the architecture of *E. coli* biofilms and hindered biofilm formation by approximately 80%. Their results indicated that the microcapsules decreased the transcriptional activity of numerous virulence-related genes that are involved in QS, thereby inhibiting biofilm formation [93]. *E. coli* Nissle 1917 (EcN) has been widely characterized and used for many years as a probiotic. This strain was able to outcompete *E. coli*, *S. aureus* and *S. epidermidis* during co-incubation [113,114], via the extracellular function of a periplasmic protein [113]. Lastly, Chen et al. [12] demonstrated that silicone pre-coated with EcN biofilms reduced the adherence of *Ent. faecalis* by 2 Log CFU for 11 days (Table 2). It can be concluded that controlling the pathogen growth with probiotic cells depends on the *Lactobacillus* strains used.

Other substances, such as the collagen-binding protein (p29) [58] and lipoteichoic acid [97,98], are emerging agents in the battle against biofilms formed in biomedical settings.

Based on the reviewed studies, independently of the strategy used, the different antibiofilm substances showed a promising effect in both the prevention and control of biofilms. The most effective probiotic strains were *L. rhamnosus*, *L. plantarum*, *L. acidophilus* and *L. fermentum*. Looking at the percentages of pathogen reduction presented in Table 1, Table 2 and Table 3, we believe that the best approach for displacement and exclusion strategies should include the use of probiotic cells, since they were responsible for higher biofilm reductions when compared to probiotic substances, whereas for the competition strategy biosurfactants caused higher reductions, along with cells and EPS. Overall, biosurfactants, EPS and cells seem to be the most effective agents to decrease biofilm formation on medical devices, regardless of the strategy used. Nevertheless, avoiding the initial attachment of pathogens by coating the surfaces seems to be the best approach to fight biofilm-based infections instead of removing established biofilms.

### 3.2. Quality Assessment

It is important to qualitatively analyze the methodologies and procedures used in the reviewed studies in order to guarantee the validity of the results and their predictive power [50]. The 45 included studies were scored according to the adapted MINORS scale presented in Table 4.

The score of the studies varied between 14 and 24, and the mean value was 19.7 ± 2.7. All papers clearly stated the aim of the work (criterion 1, mean = 1.93) and the methodologies used (criterion 4, mean = 1.84). Additionally, 44 of the 45 studies had an adequate control group, corresponding to untreated biofilms (criterion 3, mean = 1.93). All papers described the biofilm platform used, while 7 studies did not mention the surface for biofilm formation (criterion 9, mean = 1.84). Moreover, in 87% of the studies, the concentration of the antibiofilm substance was reported (criterion 6, mean = 1.87), and only one study did not report the biofilm formation period (criterion 8, mean = 1.76). Therefore, the high score attributed to these criteria indicates the methodological quality of the eligible studies.

Although these results are very encouraging, there is a lack of detail in the description of some methodological aspects. About 31% of the studies did not mention the number of replicates or independent assays (criterion 2, mean = 1.64), thus decreasing the validity of their results. One-third of the studies did not report the concentration of the pathogenic microorganisms used for biofilm inoculation (criterion 5, mean = 1.64), which is crucial to replicate the experiments, and in 51% of the studies the cell density used was not representative of an ideal clinical scenario (criterion 10, mean = 1.07), decreasing the studies’ predictive value. The different concentrations used through the studies may also contribute to increased variability, since starting from different cell concentrations will affect the biofilm treatment differently. Moreover, culture conditions were not properly reported in 73% of the studies (criterion 7, mean = 1.27). About 64% of the studies did not report the hydrodynamic conditions, but among the studies in which this parameter was mentioned, 24% were performed under agitation and 11% in static conditions. Since shear forces affect the formation and structure of biofilms on medical devices [127,128], it is essential to conveniently describe the hydrodynamic conditions used for biofilm assays.

About 36% of the included studies reported the effect of antibiofilm substances as the ratio between control and treated biofilm (criterion 11, mean = 1.58); however, this ratio does not illustrate the real degree of biofilm inhibition. It should also be noted that 42% of the studies either did not perform any statistical analysis or the statistical tests were not adequate to validate the main outcomes (criterion 12, mean = 1.31).

Although indwelling medical devices may differ in design and material, the rate and extent of biofilm formation are mainly affected by the physicochemical properties of the surface (such as hydrophobicity and roughness), number and type of microorganisms in the liquid to which the device is exposed, as well as by the biofilm formation period and the flow rate and nutrient composition of the liquid through the device [1,2,14]. Despite the promising results of probiotics against microbial biofilms in medical devices, it is important to standardize the in vitro testing conditions in order to facilitate the comparison between studies and increase their predictive value.

**Figure 3:**
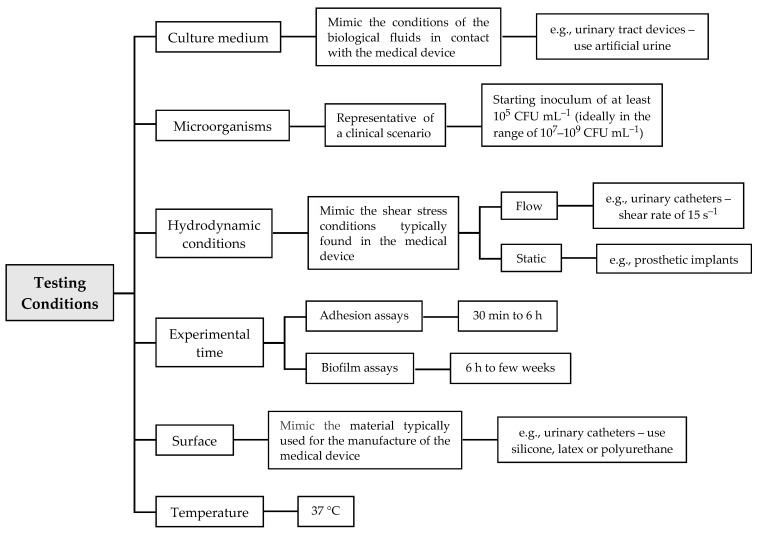
Proposal of testing conditions to be adopted in future studies.

Based on the MINORS scale (Table 4), some optimal testing conditions should be taken into consideration in future studies, which may help to systematize the results (Figure 3). Those conditions will always depend on the medical device in hand and the environment where it is inserted. The nutritional conditions (i.e., the culture medium) should mimic the biological fluids with which the device will be in contact when placed on the patient. For example, for tests of urinary tract devices, artificial urine medium is recommended [13]. Additionally, the experiments should start with an exponential phase culture, and the concentration of the starting inoculum should be of at least 10^5^ CFU mL^−1^ (ideally in the range of 10^7^–10^9^ CFU mL^−1^) in order to obtain the minimum required 3–4 Log reduction of viability or growth typical of standard tests of microbial activity [13]. Since flow conditions may affect cell adherence and biofilm formation—and, subsequently, the action of antibiofilm substances [13]—the hydrodynamic conditions used in the in vitro experiments should simulate those typically found in the target device. For instance, static conditions should be chosen when evaluating new antibiofilm approaches for prosthetic implants, whereas dynamic conditions are ideal to mimic the flow in urinary tract devices (shear rate of 15 s^−1^ [101]) and central venous catheters. Regarding the biofilm formation period, it should also be adapted to the specific biomedical scenario. Urinary catheters usually remain inserted in the patient for more than one day, and thus the experimental time should not be less than 24 h, which is the referenced time for biofilm maturation in urinary catheters [129]. For adhesion assays, it is reasonable to use periods from 30 min to 6 h, and for biofilm assays, from 6 h to weeks. For long-term exposures, we recommend the use of discrete periods (e.g., 24 h, 48 h or 72 h). Concerning the surface material, assays should be performed using a material currently used for the manufacture of these devices. For example, to mimic urinary catheters, silicone, latex, polyurethane and PDMS can be used, while for dental prostheses, saliva-coated titanium or hydroxyapatite discs are more indicated. Regardless of the biomedical application, the temperature of incubation should be 37 °C, because it is the average temperature of the human body.

To ensure the reproducibility of biofilm studies, at least three independent biological experiments should be performed. Moreover, each experiment should include at least two study groups, the control group with the untreated surface material and the treatment group. Finally, the authors must indicate a reference value for the concentration of cells in the biofilm, rather than presenting only the percentage of biofilm reduction, since the meaning of this percentage value is completely different with ten thousand or one million cells in the biofilm.

### 3.3. Meta-Analysis

Thirty-six of the 45 selected studies were included in the meta-analysis. The standard mean proportions of biofilm reduction and the respective standard deviation were retrieved from these studies and grouped according to the antibiofilm substance used either to inhibit or control microbial biofilms, including the biosurfactants, cells, EPS and CFS. Since the number of studies demonstrating the efficacy of bacteriocins and other antibiofilm substances was low, they were not included in the meta-analysis. Additionally, the methodology of analysis used for the biofilm quantification (CFU counting, CV method for biomass quantification, or other) was not discriminated because it only represents different means to evaluate the efficacy of antibiofilm substances.

**Figure 4:**
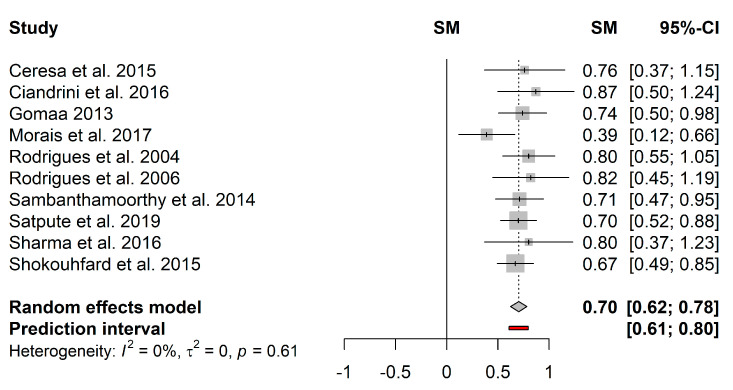
Forest plot of the pooled effect estimates for the proportion of biofilm reduction induced by biosurfactants. The vertical dashes represent the effect estimate, and the horizontal line is the respective confidence interval at 95% (95%–CI) obtained for each study. Gray squares represent the standard deviation of each study, while the gray diamond represents the pooled effect estimates. The red bar represents the predictive interval. Heterogeneity test: *I*^2^–*I*^2^ test; *τ*^2^–Tau-squared test.

The pooled effect estimates and respective 95% confidence interval were calculated for the four antibiofilm substances. Figure 4 represents the forest plot of the pooled effect estimates for the proportion of biofilm reduction induced by biosurfactants. Heterogeneity in the mean proportion of biofilm reduction was not observed among the 10 included studies (*I*^2^ = 0%; *τ*^2^ = 0; *p* = 0.61). The pooled results showed a mean proportion (95%–CI) of 70% (62%–78%) and a predictive interval of 61%–80%.

**Figure 5:**
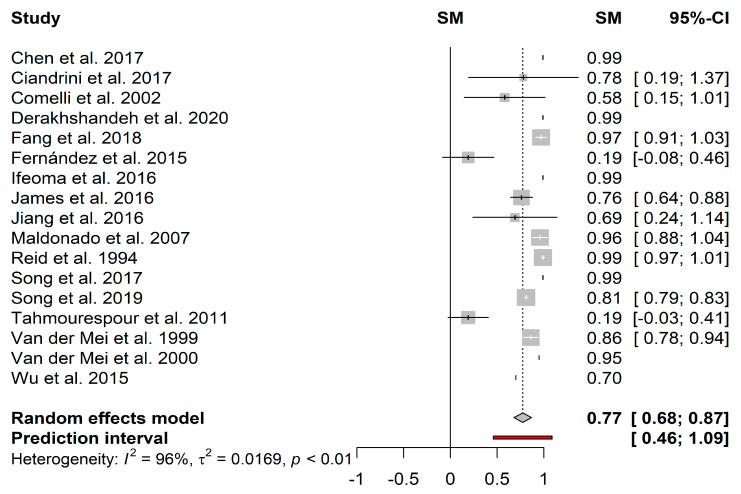
Forest plot of the pooled effect estimates for the proportion of biofilm reduction induced by cells. The vertical dashes represent the effect estimate, and the horizontal line is the respective confidence interval at 95% (95%–CI) obtained for each study. Gray squares represent the standard deviation of each study, while the gray diamond represents the pooled effect estimates. The red bar represents the predictive interval. Heterogeneity test: *I*^2^–*I*^2^ test; *τ*^2^–Tau-squared test.

In turn, the heterogeneity in the mean proportion of biofilm reduction induced by cells (Figure 5) among the 17 included studies was statistically significant (*I*^2^ = 96%; *τ*^2^ = 0.0169; *p* < 0.01). This heterogeneity can be justified by the great variability in the growth conditions among the selected studies. For example, Stepanović et al. [130,131] showed that the biofilm formation of some microorganisms was remarkably reduced under dynamic conditions. Since most of the studies did not indicate shear stress or shear rate values, it was not possible to fully compare the effectiveness of the different substances. The heterogeneity can be justified by the test of different species of probiotics, whose amount and diversity of metabolites may vary according to the species. The pooled results showed a mean proportion (95%–CI) of 77% (68%–87%) and a predictive interval of 46%–100% for probiotic cells.

**Figure 6:**
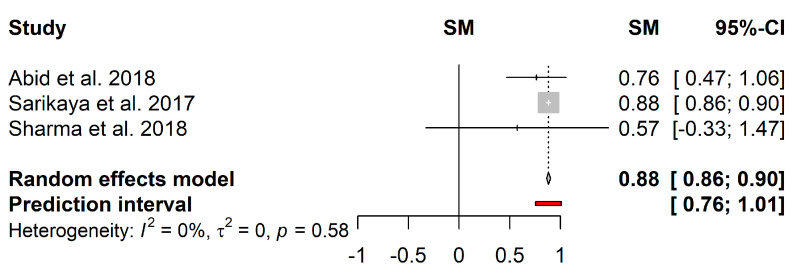
Forest plot of the pooled effect estimates for the proportion of biofilm reduction induced by EPS. The vertical dashes represent the effect estimate, and the horizontal line is the respective confidence interval at 95% (95%–CI) obtained for each study. Gray squares represent the standard deviation of each study, while the gray diamond represents the pooled effect estimates. The red bar represents the predictive interval. Heterogeneity test: *I*^2^–*I*^2^ test; *τ*^2^–Tau-squared test.

Regarding the biofilm reduction induced by EPS, only 3 studies were included in the meta-analysis (Figure 6). Although the heterogeneity in the mean proportion was not statistically significant (*I*^2^ = 0%; *τ*^2^ = 0; *p* = 0.58), these could have been caused by the reduced number of included studies. The pooled results showed a mean proportion (95%–CI) of 88% (86%–90%) and a predictive interval of 76%–100%.

**Figure 7:**
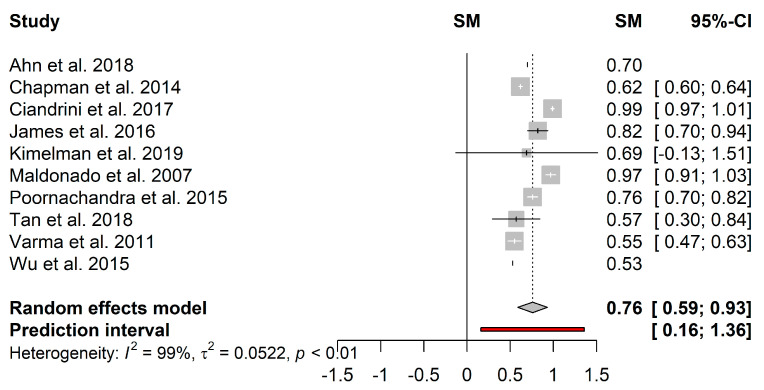
Forest plot of the pooled effect estimates for the proportion of biofilm reduction induced by cell-free supernatants. The vertical dashes represent the effect estimate, and the horizontal line is the respective confidence interval at 95% (95%–CI) obtained for each study. Gray squares represent the standard deviation of each study, while the gray diamond represents the pooled effect estimates. The red bar represents the predictive interval. Heterogeneity test: *I*^2^–*I*^2^ test; *τ*^2^–Tau-squared test.

Figure 7 presents the forest plot of the pooled effect estimates for the proportion of biofilm reduction induced by CFS. Heterogeneity in the mean proportion of biofilm reduction among the 10 included studies was statistically significant (*I*^2^ = 99%; *τ*^2^ = 0.05; *p* < 0.01). Studies’ heterogeneity can be justified by the great variability in the growth media used, which may have activated several metabolic pathways and, consequently, the quantity and diversity of produced metabolites. Kimelman and Shemesh [111] found that less *S. aureus* biofilm was formed in De Man, Rogosa and Sharpe medium than in Lysogeny broth. Furthermore, the ability of LAB to use a wide range of substrates by several pathways may enhance the heterogeneity [132]. On the other hand, the supernatants are subjected to purification procedures in which, depending on the adopted parameters (g force and time of centrifugation, type of filters), different compounds can be obtained, which may also contribute to the high heterogeneity of the studies. The pooled results displayed a mean proportion (95%–CI) of 76% (59%–93%) and a predictive interval ≥ 16%.

#### 3.3.1. Publication Bias

For the assessment of the publication bias, the Begg’s funnel plot and the Egger’s test were used (Figure S1 in Supplementary Materials). Both the funnel plot and the Egger’s test for the analyzed substances, including the biosurfactants, cell, EPS and CFS, were not statistically significant (*p* = 0.412, *p* = 0.321, *p* = 0.072, *p* = 0.875, respectively), suggesting no publication bias in sample size.

Overall, the systematic review and meta-analysis showed that the use of probiotics is a promising approach to prevent biofilm formation by a broad spectrum of pathogenic microorganisms, and their efficacy seems to be independent of the antibiofilm strategy applied: displacement, exclusion or competition. The meta-analysis results showed a pooled effect estimate for the proportion of biofilm reduction of 70% for biosurfactants, 76% for CFS, 77% for cells and 88% for EPS. Nevertheless, in the case of cells and CFS, significant heterogeneity was observed among the selected studies that must be considered when interpreting these results. Although EPS have a greater proportion of biofilm reduction, it is premature to consider that this is the most effective agent for the inhibition and control of biofilms, because only 3 studies were included, requiring further research about their biomedical application.

#### 3.3.2. Limitations and Strengths

A limitation of this meta-analysis resides in the fact that the studies were grouped regardless of the antibiofilm strategy employed (competition, displacement or exclusion) due to the high heterogeneity between the efficacy of the different antibiofilm substances. Additionally, the proportion of biofilm reduction was analyzed without considering the biofilm-forming pathogens (Gram-positive and Gram-negative bacteria, yeast or multi-species cultures). However, according to the linear regression models, the effect estimate for the proportion of biofilm reduction was not significantly influenced by the antibiofilm strategy nor by the biofilm-forming pathogen (Figure S2 in Supplementary Materials). Moreover, according to Begg’s funnel plot and Egger’s test, there was no publication bias in sample size, not influencing the meta-analysis results. Moreover, the meta-analysis results should be interpreted with caution, since about one-third of the included studies reported the effect of antibiofilm substances as the proportion of biofilm reduction, without specifying cell concentrations either for control or treated samples. Therefore, although the present meta-analysis supports the use of probiotics against microbial biofilms, it also emphasizes the need for a more comprehensive reporting of the experimental conditions and obtained results, as well as the need for more robust statistical analysis.

## 4. Conclusions

The systematic review and meta-analysis showed that the use of probiotics and their metabolites is a promising approach to reduce the impact of biofilms in medical devices. Although their efficacy seems to be independent of the antibiofilm strategy applied and the model pathogens used for testing, the prevention of initial cell attachment by coating the surfaces with probiotic biofilms was shown to be more effective than battling pre-formed pathogenic biofilms. This review addresses the need to standardize in vitro testing conditions and data analysis to facilitate the comparison between studies, thus increasing their predictive value.

## Figures and Tables

**Table 4:** Methodological index for in vitro studies and the respective mean of all studies.

Criterion	Mean
**1. A clearly stated aim:** The hypothesis/aim of the study is explicitly stated and testable by statistical means.	1.93
**2. Detection of bias:** Data were collected according to an established protocol. At least 3 independent experiments were performed for each assay.	1.64
**3. An adequate control group:** There is a control group corresponding to untreated biofilms.	1.93
**4. Appropriate methodology:** Description and explanation of the methods in accordance with the desired outcomes. The used methods are the same for control and exposure treatment.	1.84
**5. Pathogens description:** The pathogens species and quantity used for inoculation are described. 0: not reported 1: organism species OR organism quantity 2: organism species AND organism quantity	1.64
**6. Antibiofilm substances:** Description of substances used to control/prevent biofilm formation, including identity/origin and concentration. 0: not reported 1: description of origin OR concentration 2: description of origin AND concentration	1.87
**7. Culture conditions:** Description of how assays were performed in sufficient detail to repeat (or detailed methodology is referenced), including culture medium, hydrodynamic conditions and temperature. 0: not described 1: sufficient detail to repeat OR a description of culture medium OR hydrodynamic conditions OR temperature 2: sufficient detail to repeat AND a description of culture medium AND hydrodynamic conditions AND temperature	1.27
**8. Biofilm formation period:** Because some microorganisms may grow/act slower, longer incubation periods may be needed to ensure successful biofilm inhibition. 0: duration of exposure not reported 1: culture of < 6 h 2: culture of ≥ 6 h	1.76
**9. Surface:** Description of substratum for biofilm formation. 0: not described 1: description of surface OR biofilm platform 2: description of surface AND biofilm platform	1.84
**10. Predictive value:** In vitro studies may use inoculum concentrations exceeding those encountered in a clinical scenario. 0: not described 1: inoculation with flora at the same concentration as that found in clinical scenario (<10^5^ CFU/mL) 2: inoculation with a concentration of bacteria that exceeds that found in clinical scenario (>10^5^ CFU/mL)	1.07
**11. Results clarity:** The results of the study are presented in a clear and organized way. 0: results are not clear 1: results are clear 2: results are clear and easy to understand AND cell concentrations or optical density values either for control or treatment experiments were reported	1.58
**12. Adequate statistical analyses:** Description and implementation of statistical tests appropriate to the dataset, with the calculation of confidence intervals and *p* values.	1.31

CFU, Colony-Forming Units.

## Supplementary Materials

The following are available online at https://www.mdpi.com/2076-2607/9/1/27/s1, Figure S1: Begg’s funnel plot for evaluation of the publication bias in the selected studies for the four anti-biofilm substances, Figure S2: Linear regression model between the proportion of biofilm reduction and the type of anti-biofilm substance.
