# Supplementary material for: The Use of Probiotics to Fight Biofilms in Medical Devices: A Systematic Review and Meta-Analysis
Source: Microorganisms. 2020 Dec 23;9(1):27. doi: 10.3390/microorganisms9010027 (PMC7824608; doi:10.3390/microorganisms9010027)
Supplement: Supplementary file 1 [file microorganisms-09-00027-s001.pdf]

Supplementary Material

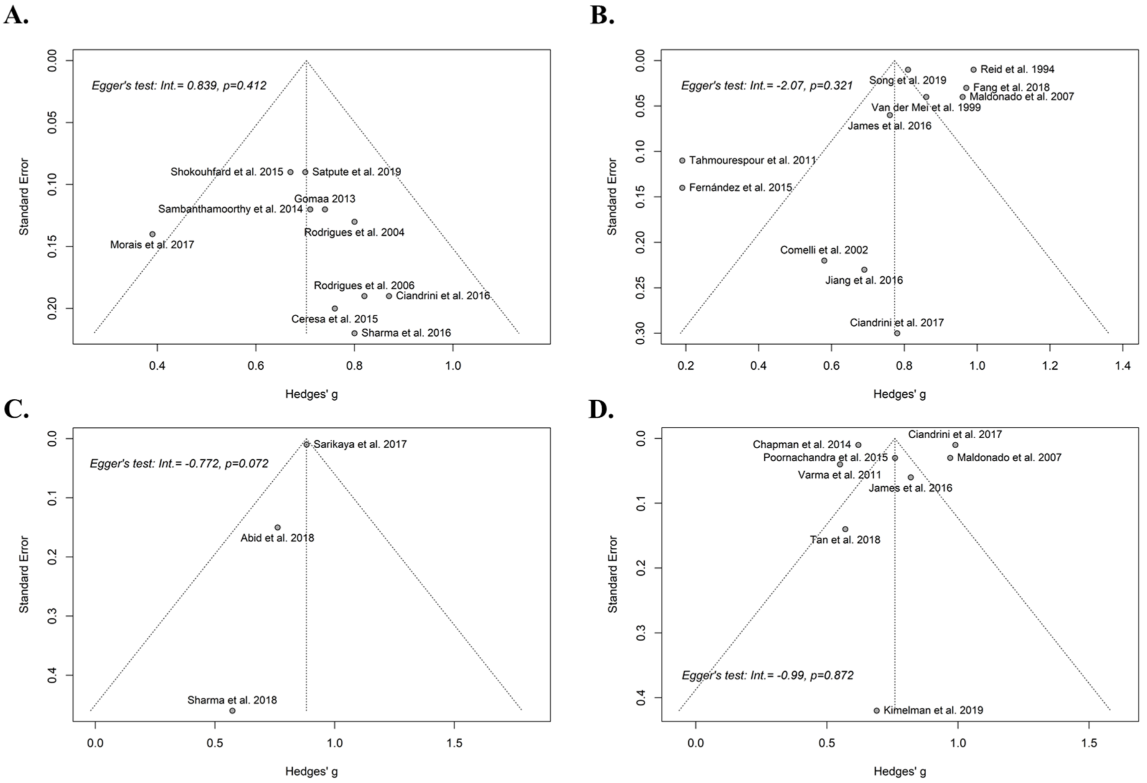

Figure S1. Begg's funnel plot for evaluation of the publication bias in the selected studies for the four anti-biofilm substances: (A) biosurfactants, (B) cells, (C) EPS, and (D) cell-free supernatants. The funnel graph plots the Hedges' g (standard mean of proportion of biofilm reduction) against the standard error. The Egger test for publication bias was not statistically significant for the four represented substances (p = 0.412, p = 0.321, p = 0.072, p = 0.875, respectively), suggesting that there was no significant publication bias associated with different sample sizes. The dashed line represents Egger's test regression.

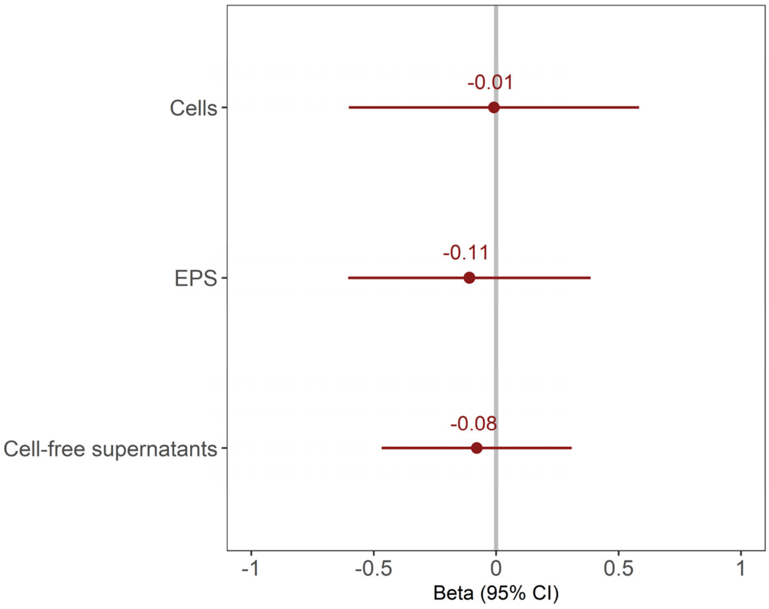

Figure S2. Linear regression model between the proportion of biofilm reduction and the type of anti-biofilm substance. Biosurfactants were used as the reference category. Models were adjusted for strategy and the biofilm-forming pathogen.
